# Supplementary material for: RPL3L-containing ribosomes determine translation elongation dynamics required for cardiac function
Source: Nat Commun. 2023 Apr 20;14:2131. doi: 10.1038/s41467-023-37838-6 (PMC10119107; doi:10.1038/s41467-023-37838-6)
Supplement: Supplementary file 1 — Supplementary Information [file 41467_2023_37838_MOESM1_ESM.pdf]

Supplementary Information

RPL3L-containing ribosomes determine translation elongation dynamics required for cardiac function

Chisa Shiraishi, Akinobu Matsumoto, Kazuya Ichihara, Taishi Yamamoto, Takeshi Yokoyama, Taisuke Mizoo, Atsushi Hatano, Masaki Matsumoto, Yoshikazu Tanaka, Eriko Matsuura-Suzuki, Shintaro Iwasaki, Shouji Matsushima, Hiroyuki Tsutsui and Keiichi I. Nakayama

SUPPLEMENTARY FIGURES

Supplementary Figures

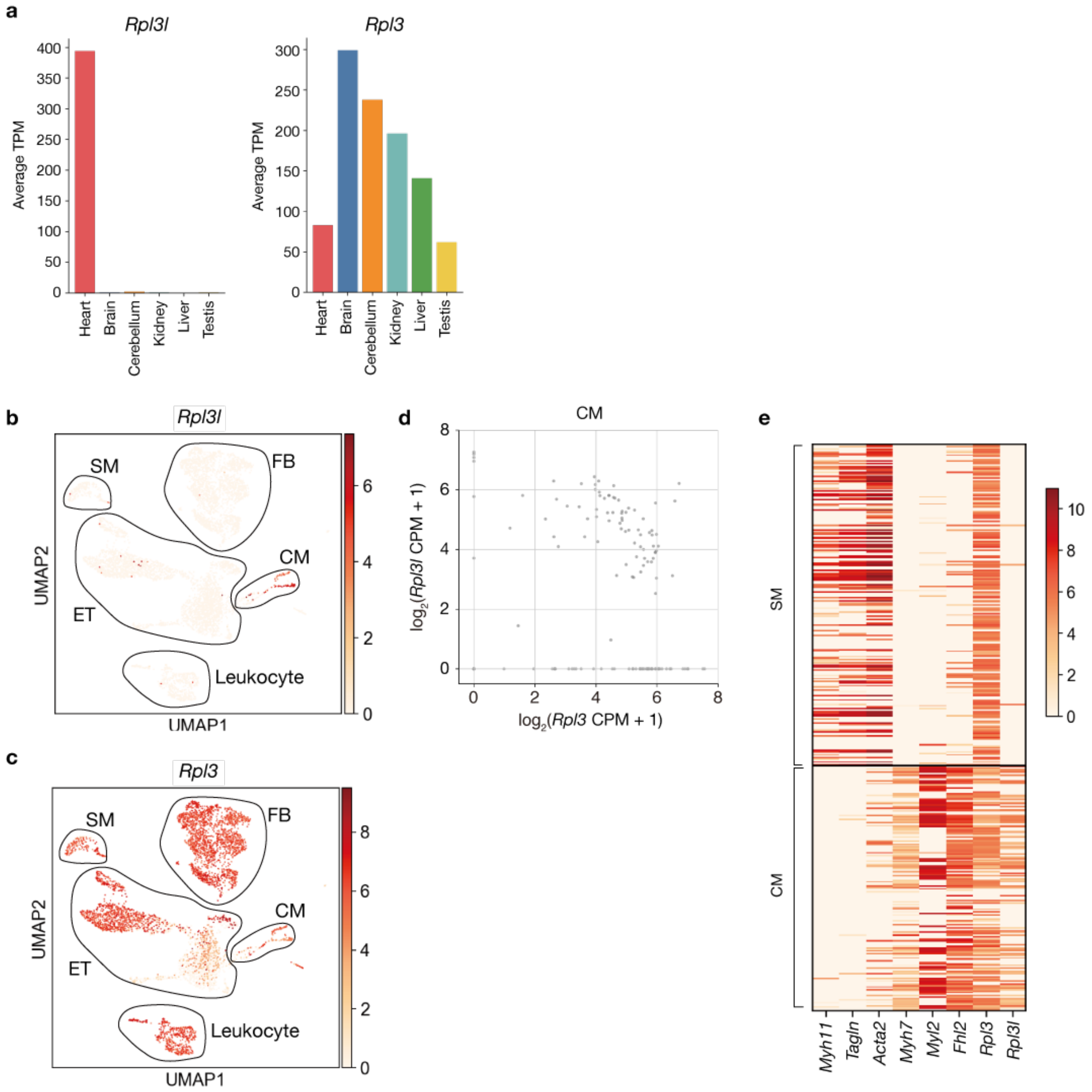

Shiraishi *et al.* Supplementary Figure 1

Supplementary Fig. 1 Expression pattern of *Rpl3l* in mouse. a, Expression patterns of *Rpl3l* and *Rpl3* in mouse tissues as determined by RNA-seq analysis. Data are from Expression Atlas and the title of experiment is "RNA-seq of 6 tissues from *Mus musculus* to investigate the evolution of gene expression levels in mammalian organs." **b**, **c**, UMAP plots from Seurat analysis of scRNA-seq data for *Rpl31* (**b**) and *Rpl3* (**c**) expression in the mouse heart. Groups corresponding to cardiomyocytes (CM), smooth muscle (SM), fibroblasts (FB), leukocytes, and endothelial cells (ET) are enclosed in black frames. Data are from *Tabula Muris*. **d**, Expression levels of *Rpl3l* and *Rpl3* in single cells for cardiomyocytes as in b and c. **e**, Heat map showing row-scaled expression of the marker genes for CM and SM as well as of *Rpl3* and *Rpl3l* in the CM and SM clusters in **b** and **c**. Source data are provided as a Source Data file.

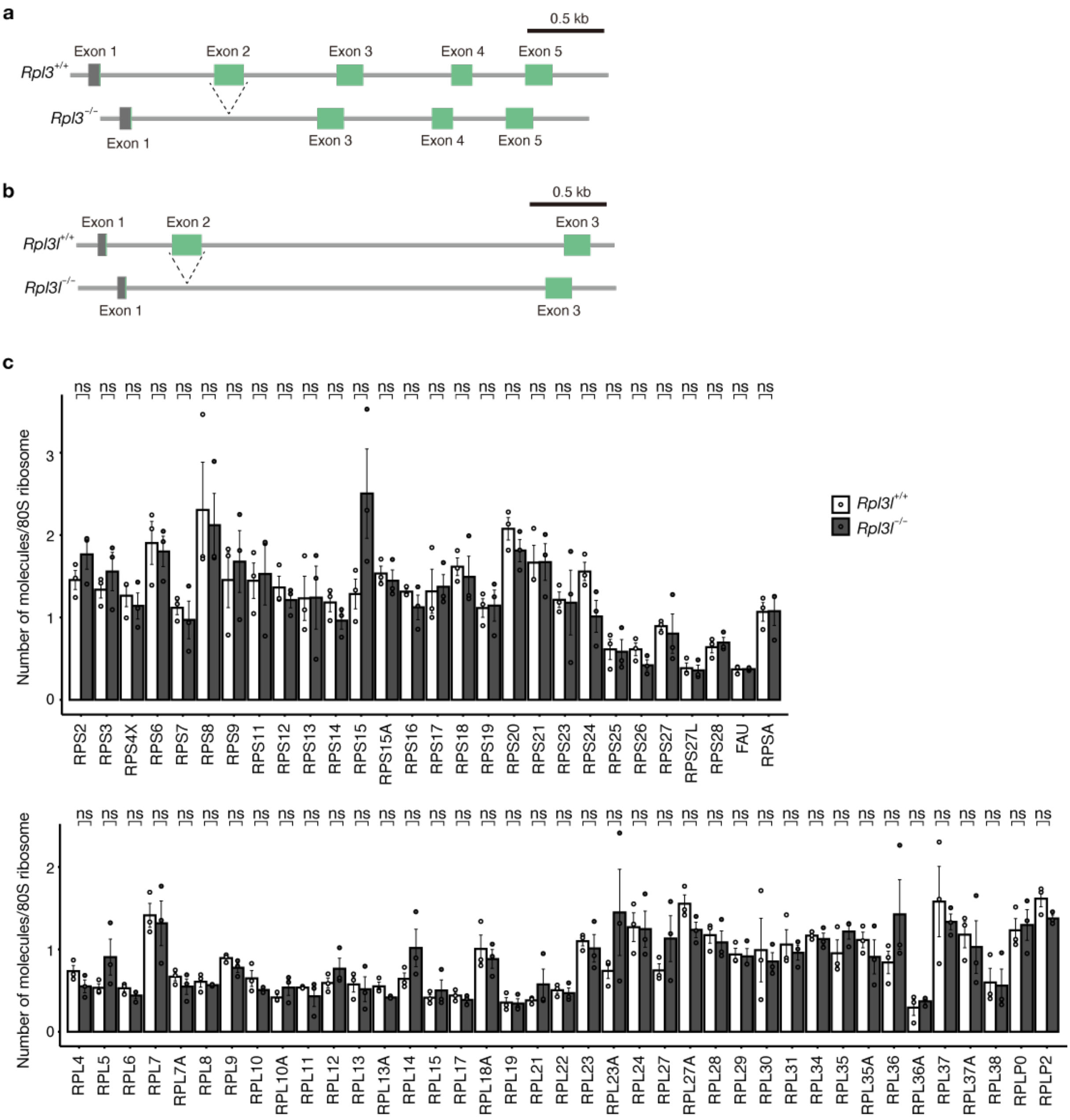

Shiraishi *et al.* Supplementary Figure 2

Supplementary Fig. 2 Generation and analysis of RPL3L knockout mice. a, **b**, Schematic representation of wild-type and mutant alleles for mouse *Rpl3* (**a**) and *Rpl3l* (**b**). Exons are denoted by boxes, and open reading frames for RPL3 and RPL3L are shown in green. **c**, Number of RP molecules other than RPL3 and RPL3L per 80S ribosome as determined by MRM analysis of the polysome fraction from the heart of *Rpl3l*$^{+/+}$ and *Rpl3l*$^{-/-}$ mice at 10 weeks of age (n = 3 mice). Data are means ± s.d. ns, two-sided Welch's t test. Source data are provided as a Source Data file.

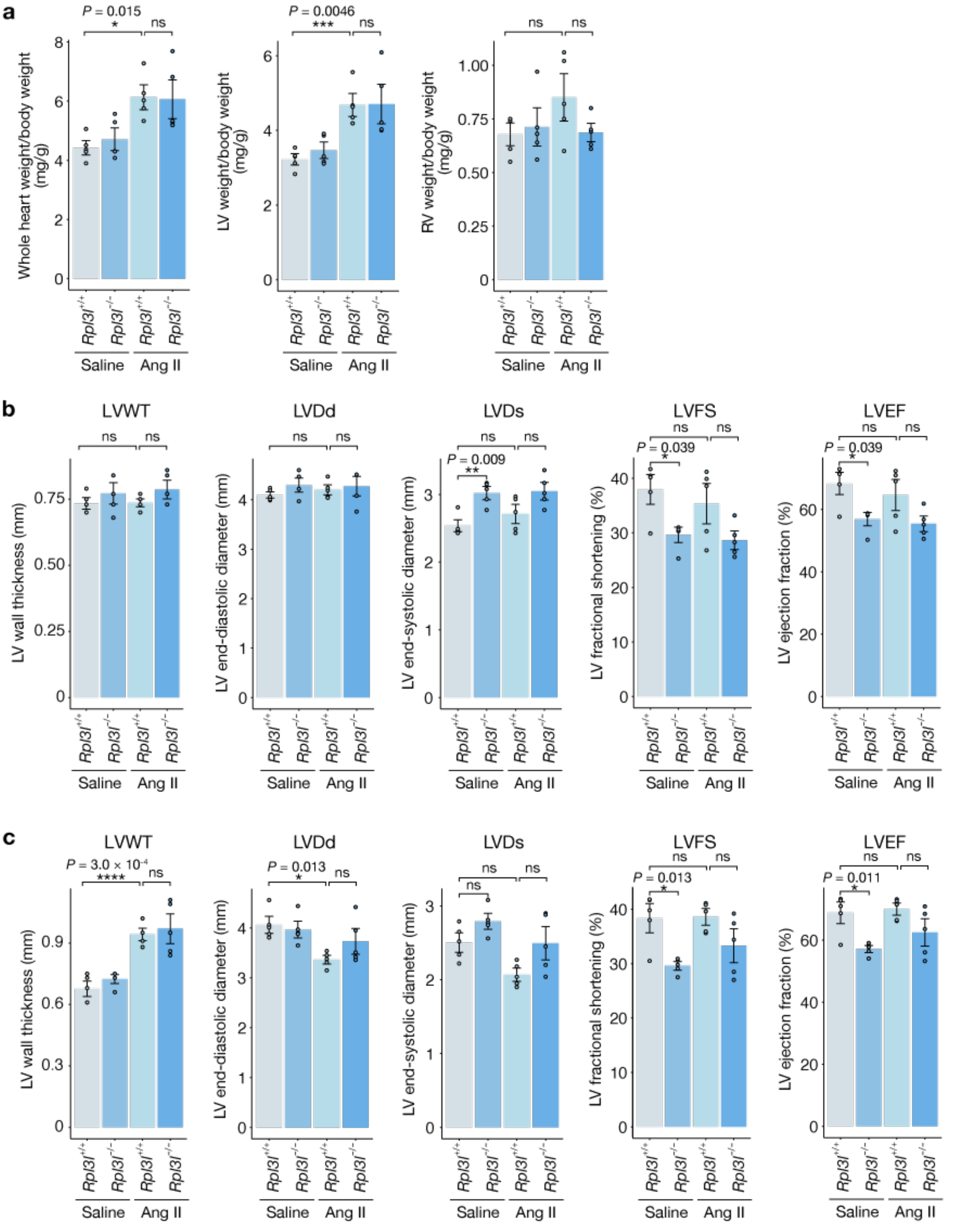

Shiraishi *et al.* Supplementary Figure 3

Supplementary Fig. 3 Cardiac pressure overloading. a, Weights of whole heart, left ventricle, and right ventricles normalized for each body weight of *Rpl3l*$^{+/+}$ and *Rpl3l*$^{-/-}$ mice (n = 5 mice) at 20 to 27 weeks of age (**e**). **b**, **c**, Quantitative analysis of LVWT, LVDd, LVDs, and LVEF for *Rpl3l*$^{+/+}$ and *Rpl3l*$^{-/-}$ mice at 18 to 19 weeks of age (n = 5 mice) before (**b**) or after ang II infusion for 7 days (**c**). All quantitative data in bar graphs are means ± s.d.

*$P < 0.05$, **$P < 0.01$, ***$P < 0.005$, ****$P < 0.001$; ns, not significant (Two-way ANOVA followed by Tukey-Kramer post-hoc multiple comparison test). Source data are provided as a Source Data file.

Shiraishi *et al.* Supplementary Figure 4

Supplementary Fig. 4 Ribo-seq analysis of RPL3L-deficient heart. a, **b**, Three-nucleotide periodicity plots (analyzed by RiboCode) and scores (analyzed by RibORF) were determined from 29-nt reads of Ribo-seq libraries prepared from heart lysates of *Rpl3l*$^{+/+}$ (**a**) and *Rpl3l*$^{-/-}$ (**b**) mice at 10 weeks of age. **c**, Relative ribosome occupancy at P- and E-site codons in the heart of *Rpl3l*$^{+/+}$ and *Rpl3l*$^{-/-}$ mice (n = 4 mice). Data were aggregated according to all codons for each amino acid. **d**, Ribosome occupancy changes at P- and E-site codons for each amino acid residue in the heart of *Rpl3l*$^{-/-}$ mice compared with that of control mice (n = 4 mice). Source data are provided as a Source Data file.

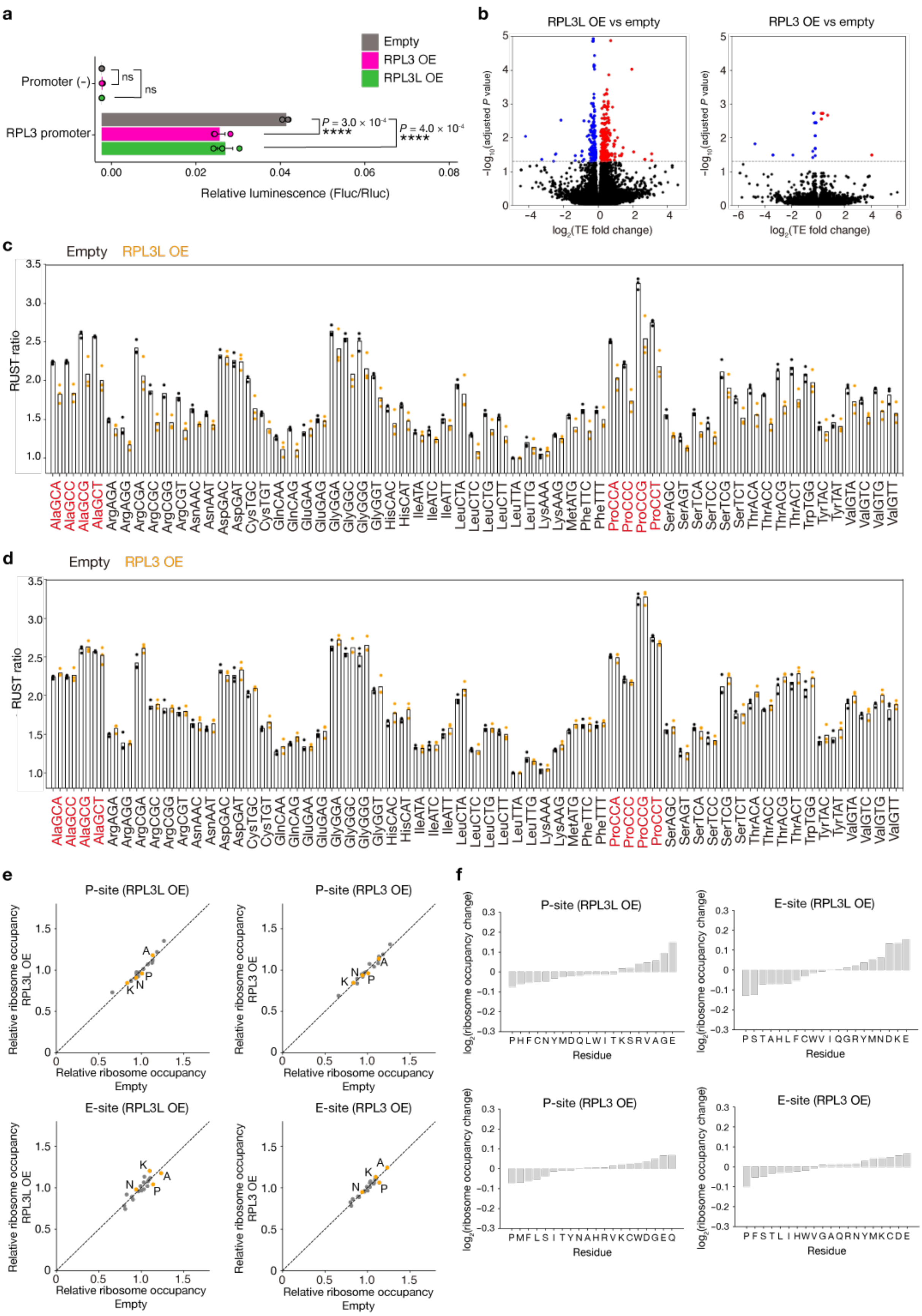

Shiraishi *et al.* Supplementary Figure 5

Supplementary Fig. 5 Analysis of C2C12 myoblasts stably expressing RPL3 or RPL3L. **a**, Luciferase assays for no promotor and RPL3 promotor constructs in RPL3L OE and RPL3 OE cells. Rluc, *Renilla* luciferase; Fluc, firefly luciferase. Assay data are means ± s.d. for three independent experiments **** $P < 0.001$; ns, not significant (two-tailed Dunnett's test). **b**, Volcano plots for differential TE. Changes in TE for C2C12 cells stably overexpressing RPL3 (RPL3 OE cells) or RPL3L (RPL3L OE cells) relative to control cells infected with the empty retrovirus were analyzed with RiboDiff ($n = 3$ biologically independent samples). P values were calculated by chi-square test and adjusted by Benjamini-Hochberg method for multiple comparisons. The gray dashed lines indicate an adjusted P value of 0.05. **c**, **d**, RUST ratio values at A-site codons in RPL3L OE (**c**) or RPL3 OE cells (**d**) compared with control cells ($n = 3$ biologically independent samples). **e**, Relative ribosome occupancy at P- and E-site codons in RPL3L OE or RPL3 OE cells ($n = 3$ biologically independent samples). Data were aggregated according to all codons for each amino acid. **f**, Ribosome occupancy changes at P- and E-site codons for each amino acid residue in RPL3L OE or RPL3 OE cells compared with control cells ($n = 3$ biologically independent samples). Source data are provided as a Source Data file.

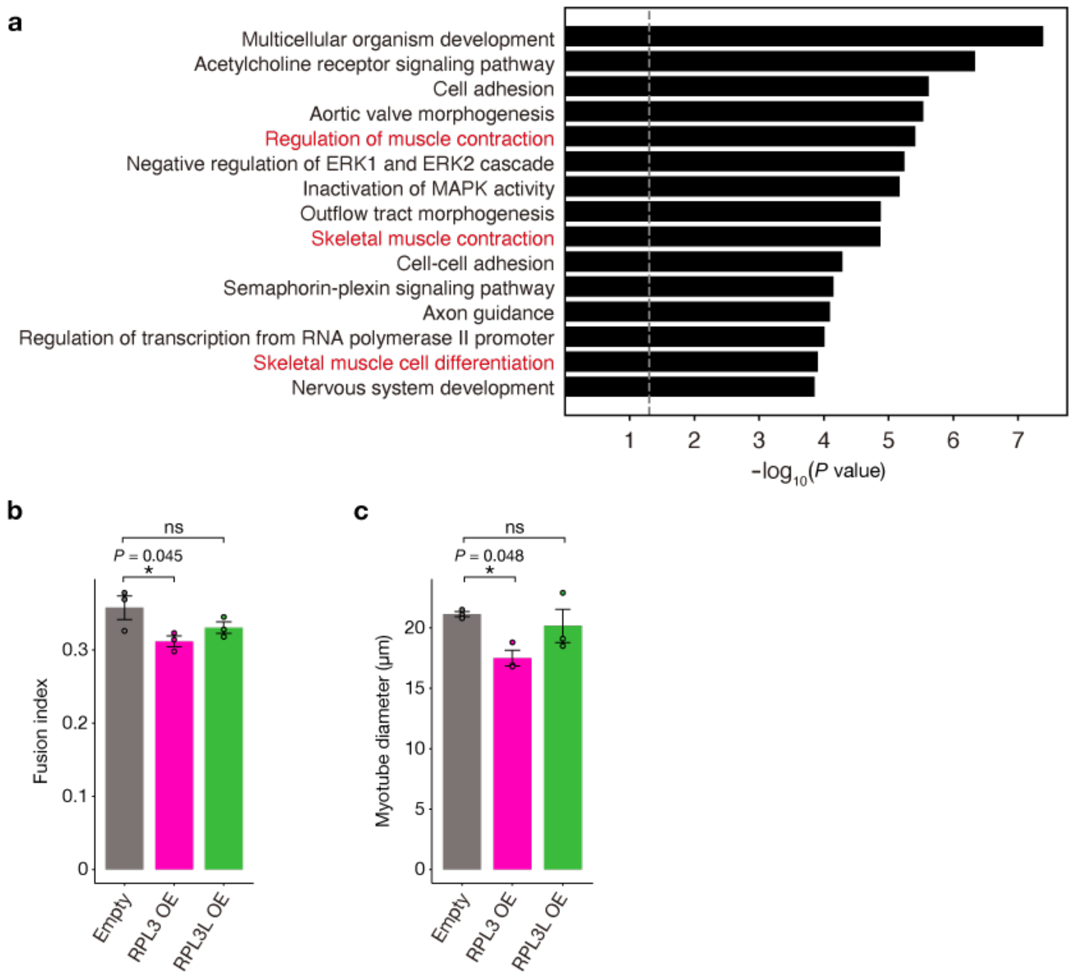

Shiraishi *et al.* Supplementary Figure 6

Supplementary Fig. 6 Myotube diameter and fusion index of C2C12 cells stably expressing RPL3 or RPL3L. a, GO analysis of genes upregulated in RPL3L OE cells compared with RPL3 OE cells. *P* values were calculated as EASE scores, modified Fisher's exact *P* values. The 15 GO terms with the smallest *P* values are listed. **b**, **c**, The fusion index (**b**) and myotube diameter (**c**) after induction of differentiation of RPL3 OE, RPL3L OE and control cells for 4 days. Assay data are means ± s.d. for three independent experiments $*P <$ 0.05; ns, not significant (two-tailed Dunnett's test). Source data are provided as a Source Data file.

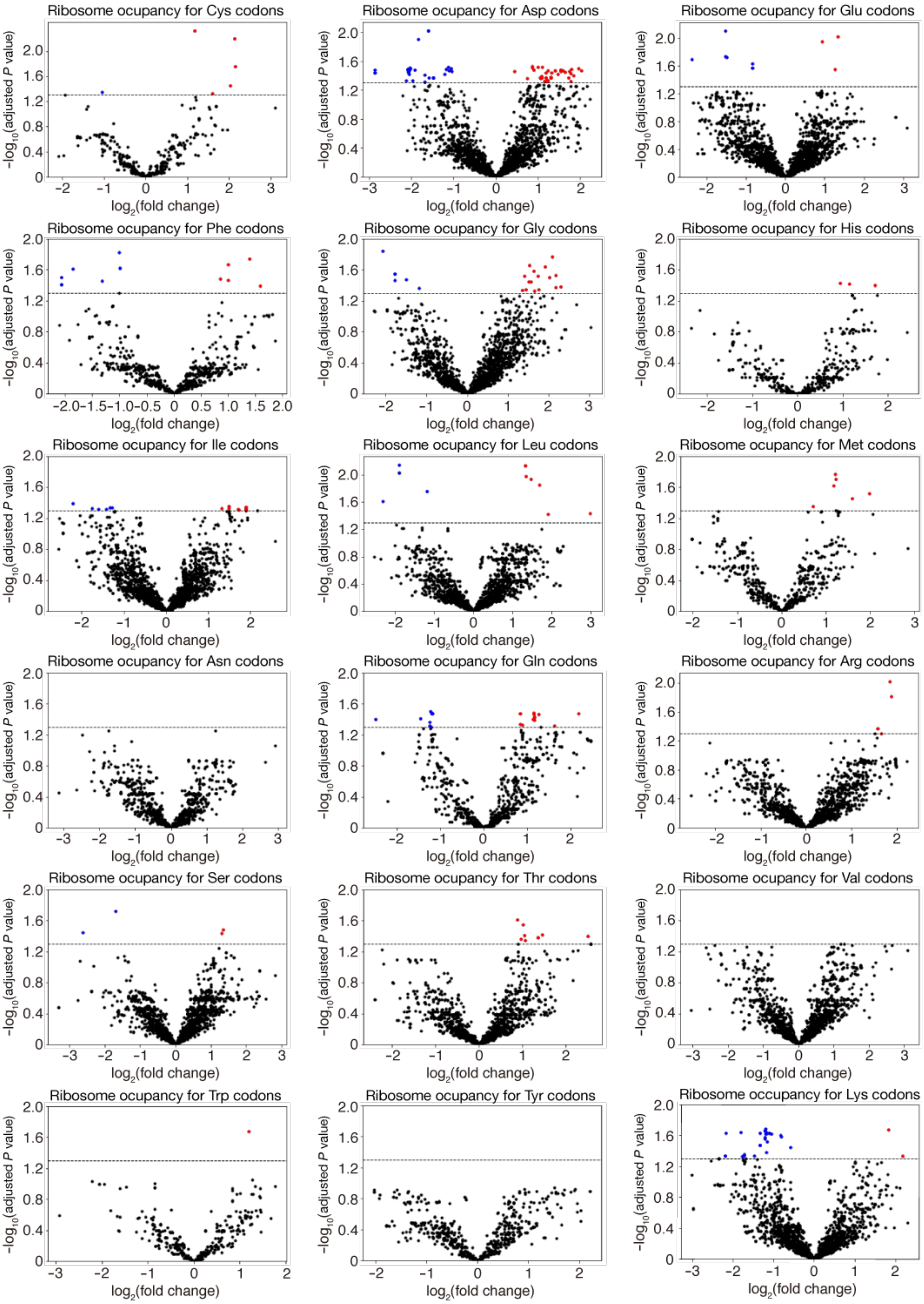

Shiraishi *et al.* Supplementary Figure 7

Supplementary Fig. 7 Ribosome occupancy at codons other than those for Pro and Ala in the RPL3L-deficient heart. Volcano plots for differential ribosome occupancy at codons other than those for Pro and Ala are shown for the heart of *Rpl3l*$^{-/-}$ mice compared with that of control mice (*n* = 4 mice). *P* values were calculated by two-sided Student's *t* test and adjusted by Benjamini-Hochberg method for multiple comparisons, and the gray dashed lines indicate an adjusted *P* value of 0.05.
