# Supplementary material for: RPL3L-containing ribosomes determine translation elongation dynamics required for cardiac function
Source: Nat Commun. 2023 Apr 20;14:2131. doi: 10.1038/s41467-023-37838-6 (PMC10119107; doi:10.1038/s41467-023-37838-6)
Supplement: Supplementary file 3 — Description of Additional Supplementary Files [file 41467_2023_37838_MOESM3_ESM.pdf]

Inventory of Additional Supplementary Files

File name: Supplementary Data 1.
Description: Echocardiographic profiles for the heart of *Rpl3l*$^{+/+}$ and *Rpl3l*$^{-/-}$ mice.

File name: Supplementary Data 2.
Description: TE values for the heart of *Rpl3l*$^{+/+}$ and *Rpl3l*$^{-/-}$ mice.

File name: Supplementary Data 3.
Description: TE values for RPL3 OE, RPL3L OE, and empty control C2C12 cells.

File name: Supplementary Data 4.
Description: Fold change in ribosome occupancy for Pro or Ala codons in the heart of *Rpl3l*$^{-/-}$ mice compared with that of control mice.

File name: Supplementary Data 5.
Description: MaxLFQ label-free quantification values for proteins in the heart of *Rpl3l*$^{+/+}$ and *Rpl3l*$^{-/-}$ mice.

File name: Supplementary Data 6.
Description: RPM values for Charged-DM-tRNA-seq analysis of the heart of *Rpl3l*$^{+/+}$ and *Rpl3l*$^{-/-}$ mice.

File name: Supplementary Data 7.
Description: TPM values for m6A-seq analysis of the heart of *Rpl3l*$^{+/+}$ and *Rpl3l*$^{-/-}$ mice.

File name: Supplementary Data 8.
Description: RPM values for Disome-seq analysis of the heart of *Rpl3l*$^{+/+}$ and *Rpl3l*$^{-/-}$ mice.

File name: Supplementary Data 9.
Description: Oligonucleotides used in this study.

File name: Supplementary Data 10.
Description: Concatemer sequences used in MRM analysis.

File name: Supplementary Data 11.
Description: Artificial protein sequences and corresponding MRM assay information.

File name: Supplementary Movie 1.
Description: Two-dimensional targeted M-mode images of the heart of *Rpl3l*$^{+/+}$ and *Rpl3l*$^{-/-}$ mice.
