# Supplementary material for: RPL3L-containing ribosomes determine translation elongation dynamics required for cardiac function
Source: Nat Commun. 2023 Apr 20;14:2131. doi: 10.1038/s41467-023-37838-6 (PMC10119107; doi:10.1038/s41467-023-37838-6)
Supplement: Supplementary file 16 — Reporting Summary [file 41467_2023_37838_MOESM16_ESM.pdf]

nature portfolio

Corresponding author(s): Keiichi I. Nakayama

Last updated by author(s): Mar 16, 2023

Reporting Summary

Nature Portfolio wishes to improve the reproducibility of the work that we publish. This form provides structure for consistency and transparency in reporting. For further information on Nature Portfolio policies, see our Editorial Policies and the Editorial Policy Checklist.

Statistics

For all statistical analyses, confirm that the following items are present in the figure legend, table legend, main text, or Methods section.

n/a	Confirmed	
☐	☒	The exact sample size (n) for each experimental group/condition, given as a discrete number and unit of measurement
☐	☒	A statement on whether measurements were taken from distinct samples or whether the same sample was measured repeatedly
☐	☒	The statistical test(s) used AND whether they are one- or two-sided<br>*Only common tests should be described solely by name; describe more complex techniques in the Methods section.*
☒	☐	A description of all covariates tested
☐	☒	A description of any assumptions or corrections, such as tests of normality and adjustment for multiple comparisons
☐	☒	A full description of the statistical parameters including central tendency (e.g. means) or other basic estimates (e.g. regression coefficient) AND variation (e.g. standard deviation) or associated estimates of uncertainty (e.g. confidence intervals)
☐	☒	For null hypothesis testing, the test statistic (e.g. F, t, r) with confidence intervals, effect sizes, degrees of freedom and P value noted<br>*Give P values as exact values whenever suitable.*
☒	☐	For Bayesian analysis, information on the choice of priors and Markov chain Monte Carlo settings
☐	☒	For hierarchical and complex designs, identification of the appropriate level for tests and full reporting of outcomes
☐	☒	Estimates of effect sizes (e.g. Cohen's d, Pearson's r), indicating how they were calculated

Our web collection on statistics for biologists contains articles on many of the points above.

Software and code

Policy information about availability of computer code

Data collection	StepOnePlus Real-Time system; QTRAP5500; KEYENCE BZ-X700; Vevo 2100 ultrasonography system; Q Exactive Hybrid Quadrupole-Orbitrap; Illumina Novaseq 6000;
Data analysis	UCSF ChimeraX; R v4.2.0; JMP16; Microsoft Excel 365; KEYENCE Hybrid Cell Count; iMPAQT-quant; DIA-NN v1.8.1; Cutadapt v1.18; FASTX-ToolKit v0.0.14; STAR v2.7.0f; UMI-tools v1.0.1; Python 3.6.8; Samtools v0.1.19; featureCounts (Subread package) v1.6.4; RibORF v1.0; RiboCode; RiboDiff v0.2.1; DESeq2 v1.36.0; RUST; HOMER v4.9.1; bedtools v2.27.1; Seurat v4.3.0; Scanpy v1.9.1; Pandas v0.25.3; Scipy v1.2.1; scikit-posthocs v0.6.7; DAVID 2021. All custom Python scripts used in the analyses of this paper are available on request.

For manuscripts utilizing custom algorithms or software that are central to the research but not yet described in published literature, software must be made available to editors and reviewers. We strongly encourage code deposition in a community repository (e.g. GitHub). See the Nature Portfolio guidelines for submitting code & software for further information.

Data

Policy information about availability of data

All manuscripts must include a data availability statement. This statement should provide the following information, where applicable:

- Accession codes, unique identifiers, or web links for publicly available datasets
- A description of any restrictions on data availability
- For clinical datasets or third party data, please ensure that the statement adheres to our policy

All sequence data have been deposited in GEO under the accession number GSE203072 (https://www.ncbi.nlm.nih.gov/geo/query/acc.cgi?acc=GSE203072). The

MS data have been deposited with the ProteomeXchange Consortium via the JPOST partner repository under the data set identifiers PXD034018 (http://proteomecentral.proteomexchange.org/cgi/GetDataset?ID=PXD034018) and PXD034019 (http://proteomecentral.proteomexchange.org/cgi/GetDataset?ID=PXD034019). Structural data for human 80S ribosome (PDB ID: 6IP5, https://www.rcsb.org/structure/6IP5) and A-tRNA (PDB ID: 4V5D, https://www.rcsb.org/structure/4V5D) were obtained from the Protein Data Bank. The predicted model of human RPL3L was obtained from the AlphaFold protein structure database (Identifier: AF-Q92910-F1, https://alphafold.ebi.ac.uk/entry/Q92901). Source data are provided with this paper.

Human research participants

Policy information about [studies involving human research participants and Sex and Gender in Research.]

Reporting on sex and gender	N/A
Population characteristics	N/A
Recruitment	N/A
Ethics oversight	N/A

Note that full information on the approval of the study protocol must also be provided in the manuscript.

Field-specific reporting

Please select the one below that is the best fit for your research. If you are not sure, read the appropriate sections before making your selection.

- [x] Life sciences
- [ ] Behavioural & social sciences
- [ ] Ecological, evolutionary & environmental sciences

For a reference copy of the document with all sections, see nature.com/documents/nr-reporting-summary-flat.pdf

Life sciences study design

All studies must disclose on these points even when the disclosure is negative.

Sample size	Sample size of each measurement was determined by the practical limitations of the protocol utilized. No statistical estimation of sample size was performed, but the sample-size were determined based on previous reports (Ikeda et al. Sci. Rep. 2015 Oct 30;5:15881., Sharma et al. Cell Rep. 2019 Mar 19;26(12):3313-3322.).
Data exclusions	No data was excluded.
Replication	All experiments were conducted with multiple biological replicates shown in figures, figure legends or methods. For almost all experiments, more than 3 biologically independent replicates were analyzed.
Randomization	All individual mice or samples were randomly allocated to the experimental groups.
Blinding	The study was essentially not blinded because it was necessary for investigators to be aware of the information of mouse genotypes, conditions and cell types to be compared.

Reporting for specific materials, systems and methods

We require information from authors about some types of materials, experimental systems and methods used in many studies. Here, indicate whether each material, system or method listed is relevant to your study. If you are not sure if a list item applies to your research, read the appropriate section before selecting a response.

Materials & experimental systems

n/a	Involved in the study	
	x	Antibodies
	x	Eukaryotic cell lines
x		Palaeontology and archaeology
	x	Animals and other organisms
x		Clinical data
x		Dual use research of concern

Methods

n/a	Involved in the study	
x		ChIP-seq
x		Flow cytometry
x		MRI-based neuroimaging

Antibodies

Antibodies used	Mouse monoclonal anti-Myosin (Skeletal, Fast)/Sigma, M4276/1:250<br>Alexa Fluor 488–conjugated goat anti-mouse IgG/Thermo Fisher Scientific, A-11029/1:1000<br>Alexa Fluor 488–conjugated WGA/Thermo Fisher Scientific, A11261/5μg/mL<br>m6A antibody/Epigentek, P-9016/1:100
Validation	Mouse monoclonal anti-Myosin (Skeletal, Fast)/Sigma, M4276 (https://www.sigmaaldrich.com/JP/en/product/sigma/m4276)<br>Alexa Fluor 488–conjugated goat anti-mouse IgG/Thermo Fisher Scientific, A-11029 (https://www.thermofisher.com/antibody/product/Goat-anti-Mouse-IgG-H-L-Highly-Cross-Adsorbed-Secondary-Antibody-Polyclonal/A-11029)<br>Alexa Fluor 488–conjugated WGA/Thermo Fisher Scientific, A11261 (https://www.thermofisher.com/order/catalog/product/jp/ja/W11261)<br>m6A antibody/Epigentek, P-9016 (https://www.epigentek.com/catalog/n6-methyladenosine-m6a-polyclonal-antibody-p-71806.html)

Eukaryotic cell lines

Policy information about cell lines and Sex and Gender in Research

Cell line source(s)	HEK293T and C2C12 cells were obtained from ATCC. HEK293T cells are derived from a female human, and C2C12 myoblasts are derived from a female mouse.
Authentication	No additional authentication was performed by the authors.
Mycoplasma contamination	Cells were tested for mycoplasma using the MycoAlert (Lonza), and all cell lines were confirmed to be negative for mycoplasma contamination.
Commonly misidentified lines<br>(See ICLAC register)	Commonly misidentified cell line was not used.

Animals and other research organisms

Policy information about studies involving animals; ARRIVE guidelines recommended for reporting animal research, and Sex and Gender in Research

Laboratory animals	RPL3 KO mice and RPL3L KO mice (C57BL/6J strain) were generated as described in methods section and 8-21 weeks old male mice were used in our experiments. All mice were housed in the specific pathogen–free animal facility at Kyushu University in accordance with institutional guidelines under the following conditions: 22°C ambient temperature, 50–60% humidity, 12 h dark/light cycle, and free access to water and rodent chow CA-1 (CLEA Japan).
Wild animals	No wild animals were used.
Reporting on sex	All analyzed mice were male in the study, because male mice are commonly used for cardiac analysis.
Field-collected samples	No field collected samples were used in the study.
Ethics oversight	All animal experiments were approved by the animal ethics committee of Kyushu University (A20-169-0, A21-271-0, and A22-013-0) and were conducted in compliance with the university guidelines and regulations for animal experimentation.

Note that full information on the approval of the study protocol must also be provided in the manuscript.
